# Monitoring the impact of Movement Control Order (MCO) in flattening the cummulative daily cases curve of Covid-19 in Malaysia: A generalized logistic growth modeling approach

**DOI:** 10.1016/j.idm.2021.07.004

**Published:** 2021-07-21

**Authors:** Nicholas Tze Ping Pang, Assis Kamu, Mohd Amiruddin Mohd Kassim, Chong Mun Ho

**Affiliations:** a Faculty of Medicine and Health Sciences, Universiti Malaysia Sabah, Jalan UMS, 88400, Kota Kinabalu, Sabah, Malaysia; b Mathematics With Economics Programme, Faculty of Science and Natural Resources, Universiti Malaysia Sabah, Jalan UMS, 88400, Kota Kinabalu, Sabah, Malaysia

**Keywords:** COVID-19, Malaysia, Generalized logistic growth modelling, Forecast

## Abstract

**Introduction:**

COVID-19 has affected almost every country in the world, which causing many negative implications in terms of education, economy and mental health. Worryingly, the trend of second or third wave of the pandemic has been noted in multiple regions despite early success of flattening the curve, such as in the case of Malaysia, post Sabah state election in September 2020. Hence, it is imperative to predict ongoing trend of COVID-19 to assist crucial policymaking in curbing the transmission.

**Method:**

Generalized logistic growth modelling (GLM) approach was adopted to make prediction of growth of cases according to each state in Malaysia. The data was obtained from official Ministry of Health Malaysia daily report, starting from 26 September 2020 until 1 January 2021.

**Result:**

Sabah, Johor, Selangor and Kuala Lumpur are predicted to exceed 10,000 cumulative cases by 2 February 2021. Nationally, the growth factor has been shown to range between 0.25 to a peak of 3.1 throughout the current Movement Control Order (MCO). The growth factor range for Sabah ranged from 1.00 to 1.25, while Selangor, the state which has the highest case, has a mean growth factor ranging from 1.22 to 1.52. The highest growth rates reported were in WP Labuan for the time periods of 22 Nov - 5 Dec 2020 with growth rates of 4.77. States with higher population densities were predicted to have higher cases of COVID-19.

**Conclusion:**

GLM is helpful to provide governments and policymakers with accurate and helpful forecasts on magnitude of epidemic and peak time. This forecast could assist government in devising short- and long-term plan to tackle the ongoing pandemic.

## 1 Introduction

Coronavirus disease 19 (COVID-19) has inundated the whole world ever since its emergence in Wuhan, China in late 2019 (Lau et al., 2020). Over the year 2020, it has afflicted almost every country round the world, resulting in unprecedented multiple lockdowns and great consequent disruption to livelihood, education, employment, and mental health issues (Coibion et al., 2020; Kassim et al., 2021; Kumar & Nayar, 2020; Mohd Kassim, Ayu, et al., 2020; Mohd Kassim, Pang, et al., 2020; Mukhsam et al., 2020; Pang et al., 2020; Sahu, 2020). An especially worrying and puzzling trend is that it has re-emerged in multiple regions of the world in second or even third successive waves, after previous lockdowns between March to June 2020 succeeded in flattening the initial epidemiological curves (Ali, 2020; Bontempi, 2020; Looi, 2020; Middleton et al., 2020; Zainudin et al., 2020).

Hence, it is of paramount importance that epidemiologists and statisticians are able to predict trends in Covid-19 case burdens at the initial tick or upswing of future waves. The generalized logistic model (GLM) was previously developed by Richards in 1959 (Richards, 1959), and its empirical function demonstrates remarkable synchronicities with Ebola and SARS data for the purpose of disease outbreak prediction (Glennon et al., 2019; Hirose, 2007). In the context of the current pandemic, the GLM has been instrumental as an epidemiological model in predicting trends in the China mainland, Iran, Philippines and Italy for Covid-19 caseloads (Ahmadi et al., 2020; Aviv-sharon & Aharoni, 2020; Martelloni & Martelloni, 2020; Pelinovsky et al., 2020; Wang et al., 2020; Wu et al., 2020a).

Currently, Malaysia is gripped in the throes of a third more infectious and more deadly wave that originated at the tail end of a state election in the state of Sabah on 26 Sept 2020 (Sukumaran, 2020; Zainudin et al., 2020). Sabah is situated on Borneo island, together with Sarawak state and WP Labuan, and hence is only accessible by flight from all West Malaysian states. However, at that time, despite the rising case figures in Sabah, there were no movement restrictions throughout the country, and Covid-19 swabs were not required to travel from state to state. As a result, from a previous low of single digit Covid-19 cases throughout Malaysia, the number of cases gradually grew to hit the thousands, with the pandemic spread encompassing the entire nation due to the double jeopardy of uncontrollable outbreaks in detention centers and workers’ dormitories (Anand, 2020; Liew, 2021). The latter especially resulted in an overspill due to overcrowding and free access to local communities, with consequent widespread community infection. As a result, various iterations of a Conditional Movement Control Order (CMCO) was reimplemented in Malaysia in the first month of October in various states, chiefly involving Sabah, Selangor, and WP Kuala Lumpur. After a period where pandemic spread appeared to have been quelled, free movement was reinstated throughout the country in December 2020, during the year-end holidays, causing high amount of movement between states that had higher and lower infectivity (Rahman, 2021). At the current moment, Malaysia is struggling to emerge from this current wave, with case numbers in the past few weeks equaling those of the entire preceding period. Hence, it is more crucial than ever that we marry epidemiology and clinical medicine, putting current epidemiological data to good use to make instant predictions about future trends that can help national governments plan and refine policies that straddle the balance between economic insecurity and judicious lockdowns.

## 2 Methods

Official data was obtained, stratified into the 15 states and federal territories of Malaysia, from the Ministry of Health official daily statistics (Ministry of Health Malaysia, 2021), which was extracted from the official Ministry of Health website. The first day of data collection was 26 September 2020, the day on which the state election happened that triggered the national third wave of infections. Prior to the election, case loads in Malaysia had stabilized to single digits nationwide, and hence the election itself was the trigger to which case loads expanded significantly; however, due to latency in testing, case loads only began to reflect true burden of Covid-19 infection in the community two weeks after 26 September 2020. Data was obtained till 1 January 2021, prior to the imposition of a stricter movement control order in response to the unremitting third wave of Covid-19 infection. Hence, from 26 September 2020 to 1 January 2021, the nation was in the same phase of movement control, namely the Conditional Movement Control Order, with slightly differing implementations between states depending on the National Security Council instructions in each state-level administration center. The common themes of this CMCO were restriction of inter-district movement in Sabah, Selangor and WP Kuala Lumpur from October to November 2020, shutting of non-essential businesses in Sabah for the first two weeks of October, and restriction of all meetings and conventions.

Previously, logistics models were developed by Verhulst in 1838, with the express function of performing growth modelling on biological populations (Verhulst, 1838). This was extended in the 1950s by Richards et al. into *Generalized* logistic growth to account for more flexible S-shape curvatures, leading to increasing asymmetry in growth curves (Richards, 1959). There are four parameters to be estimated in the model, namely the upper asymptote or the maximum cumulative case incidence (K), the intrinsic growth rate during the exponential phase (r), the point of inflection or the turning point which is the time where maximum number of cases per day occur $\left(t_{m}\right)$, and the parameter that in part determines the point of inflection (δ). The model has been recently used by many researchers to predict the cumulative number of daily cases of Covid-19 (Aviv-sharon & Aharoni, 2020; Jain et al., 2020; Wu et al., 2020b). The cumulative cases, C at time t is estimated as follows:

Ct=K∞[1+(δ−1)e−r(t−tm)]1(1−δ)

Noise in the data contributes to uncertainty in parameter estimates within this system. In efforts to estimate uncertainty inherent in the model estimates and construct the 95 % confidence intervals, a parametric bootstrap approach was employed to randomly generate multiple samples from the best-fit curve of the empirical distribution of the parameters. For each of the states’ data sets, 1000 bootstrap iterations were computed. With these four estimated model parameters, we then estimate the cumulative cases on day 115 and 130 with 95 % prediction intervals. NCSS statistical software version 11 was used during the analysis.

Multiple assumptions were inherent. Firstly, as the number of daily cases is influenced by the daily volume of tests conducted, it was implicitly assumed that a similar number of tests were performed daily. Secondly, due to strict international travel regulations, there was little to no importation of foreign cases. Thirdly, as there were strict clampdowns on any form of mass gatherings, Covid-19 was permitted to run its natural epidemiological course; hence human behaviour did not need to be factored into the model. Fourthly, it was implied that there was continuous imposition of various national regulatory measures, including heightened personal hygiene, isolation, vigorous contact tracing, restrictions on social contacts and transportation migration, are maintained continuously.

## 3 Results

i. Comparing the number of days taken to record 10^th^, 100^th^, 1000^th^, and 10000^th^ case

**Fig. 1:**
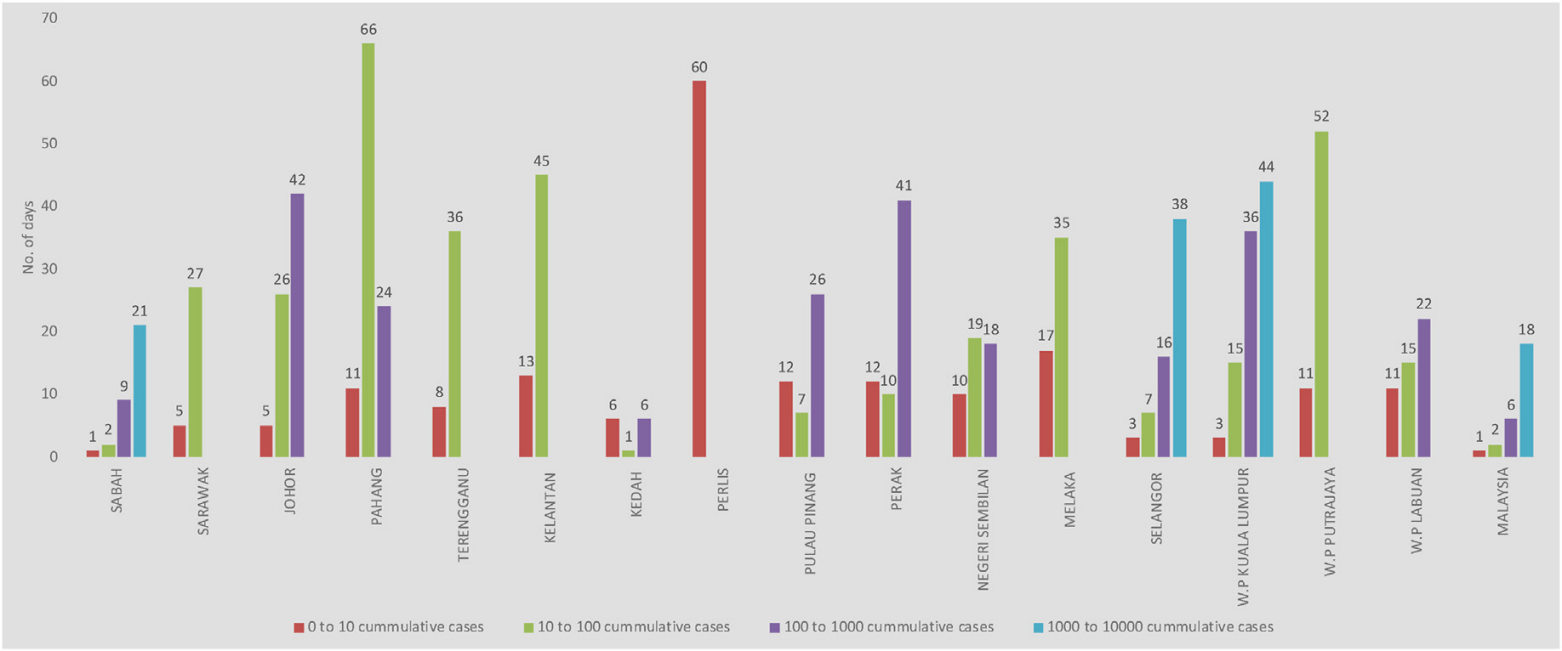
Number of days taken to record 10th, 100th, 1000th, and 10000^th^ case by state.

**Fig. 2:**
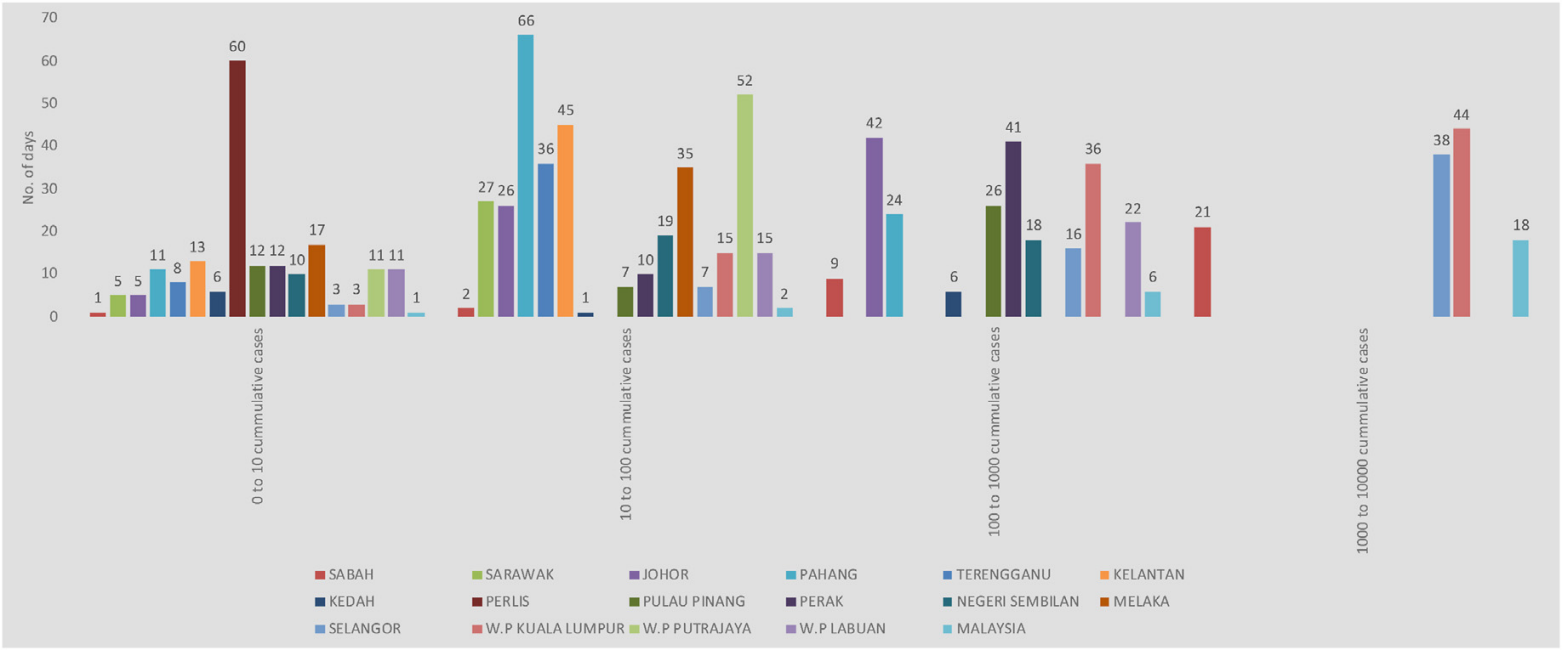
Number of days taken to record 10th, 100th, 1000th, and 10000^th^ case by case category.

Fig. 1 demonstrates the number of days it has taken each individual state to reach the next power of ten in number of cases. Higher bars at higher numbers of cases are hence the desired outcome, with the ideal outcome being a very high red bar without any successive coloured bars appearing. Perlis clearly never exceeds 100 cumulative cases, as it went 60 days without moving to the next power of 10. Most other states demonstrated corresponding increases in number of days to reach the next power of 10, with the exception of Pahang, Kedah and Negeri Sembilan. In an illustrative example, Pahang took 65 days to get from 10 to 100 cases, but subsequently only took 24 days to get from 100 to 1000 cases. Kedah also experienced an exponential rise where it took 1 day to get from 10 to 100, and only 6 days to get from 100 to 1000. Fig. 2 demonstrates the same data but collated by 10^x^ cases, demonstrating that in the time period concerned only three states – Sabah, Selangor, and WP Kuala Lumpur managed to exceed the threshold of 10000 cases.

ii. Predicting the cumulative cases using generalized logistic growth model

Table 1 demonstrates the predicted cumulative cases on Day 115 and Day 130 of the current MCO after the Sabah state election. Sabah, Johor, Selangor and WP Kuala Lumpur are the main states that are predicted to exceed 10,000 cases, with Negeri Sembilan and Melaka predicted to exceed 8000 cases by the 2 February 2021 timepoint.

**Table 1:** Predicted cumulative cases on Day 115 (18 January 2021) and Day 130 (2 February 2021) by state.

State	Day 115 (18 January 2021)			Day 130 (2 February 2021)		
	Predicted cumulative cases	Bootstrap confidence limits at 0.95 confidence level		Predicted cumulative cases	Bootstrap confidence limits at 0.95 confidence level	
	Bootstrap mean	Lower	Upper	Bootstrap mean	Lower	Upper
Sabah	37430.07	37082.83	37820.65	38691.37	38174.54	39225.96
Sarawak	382.40	374.46	389.45	382.41	374.43	389.47
Johor	12911.00	11315.10	16305.13	26781.94	18282.91	40532.44
Pahang	1328.75	828.96	1669.20	1697.94	678.64	2194.10
Terengganu	216.55	205.14	227.59	240.24	223.87	254.56
Kelantan	771.46	599.15	1004.49	1021.21	636.47	1430.42
Kedah	2580.62	2483.01	2621.19	2631.20	2506.43	2671.67
Perlis	12.87	11.39	13.27	12.92	10.32	13.29
Pulau Pinang	3973.48	3925.69	4001.10	4592.02	4526.43	4624.04
Perak	3082.74	3027.03	3174.54	3119.18	3046.95	3227.55
Negeri Sembilan	7693.87	7504.79	7860.38	8177.70	7856.62	8449.05
Melaka	2992.15	2408.84	3572.37	8077.01	5952.13	10270.33
Selangor	41534.78	40022.47	43542.67	50194.96	46279.61	54642.53
WP Kuala Lumpur	18128.14	17323.09	18785.93	25579.52	23331.73	27247.11
WP Putrajaya	196.92	189.88	206.98	224.53	215.06	239.02
WP Labuan	1565.99	1505.84	1631.77	1574.07	1503.42	1644.16
MALAYSIA	130546.86	129528.88	131515.02	157130.26	155456.37	158462.46

Table 2 shows the estimated four parameters of the model for each state. There are three states with the negative sign of inflection point (δ), which are Pahang, Terengganu, and Kedah. For a logistic model, the sign should be positive which represents a rising point of inflection. The negative sign means that the point is a falling point of inflection. In other words, the trends of cumulative cases in the states particularly during the studied period are not following a logistic pattern. Therefore, the suggested model which is generalized logistic growth model is not appropriate to be used in these three states.

**Table 2:** Parameters of the generalized logistic growth models by state.

State	K	δ	r	$t_{m}$	Pseudo R-squared
Sabah	41144.01	0.50	0.03	30.01	0.999
Sarawak	382.11	2.33	0.14	38.04	0.993
Johor	456494.42	1.38	0.03	190.11	0.996
Pahang	5925.21	−0.17	0.00	32.13	0.964
Terengganu	1270.989	−0.104	0.001	−73.005	0.963
Kelantan	7247.323	0.592	0.005	186.579	0.970
Kedah	2756.87	−1.11	0.02	−35.01	0.967
Perlis	12.82	8.51	0.14	64.91	0.919
Pulau Pinang	92742.152	0.106	0.001	201.042	0.996
Perak	3138.37	2.22	0.09	69.77	0.998
Negeri Sembilan	8976.12	0.74	0.03	57.01	0.998
Melaka	12357.10	6.09	0.34	131.20	0.954
Selangor	78959.31	1.16	0.02	98.94	0.998
WP Kuala Lumpur	257385.12	0.79	0.01	216.93	0.997
WP Putrajaya	4116.351	0.071	0.001	136.965	0.976
WP Labuan	1576.30	0.62	0.06	33.95	0.991
MALAYSIA	2397160.2	0.4	0.0	306.3	0.999

Note: Day 1 is 25 Sep 2020 and the predicted cumulative cases were estimated using a generalized logistic growth model.

Growth factor is defined as follows:

$$G_{t+1}=\frac{f_{t+1}-f_{t}}{f_{t}-f_{t-1}}$$

where $f_{t}$ is the total cases at time t, and $G_{t+1}$ is the growth factor at time t.

**Fig. 3:**
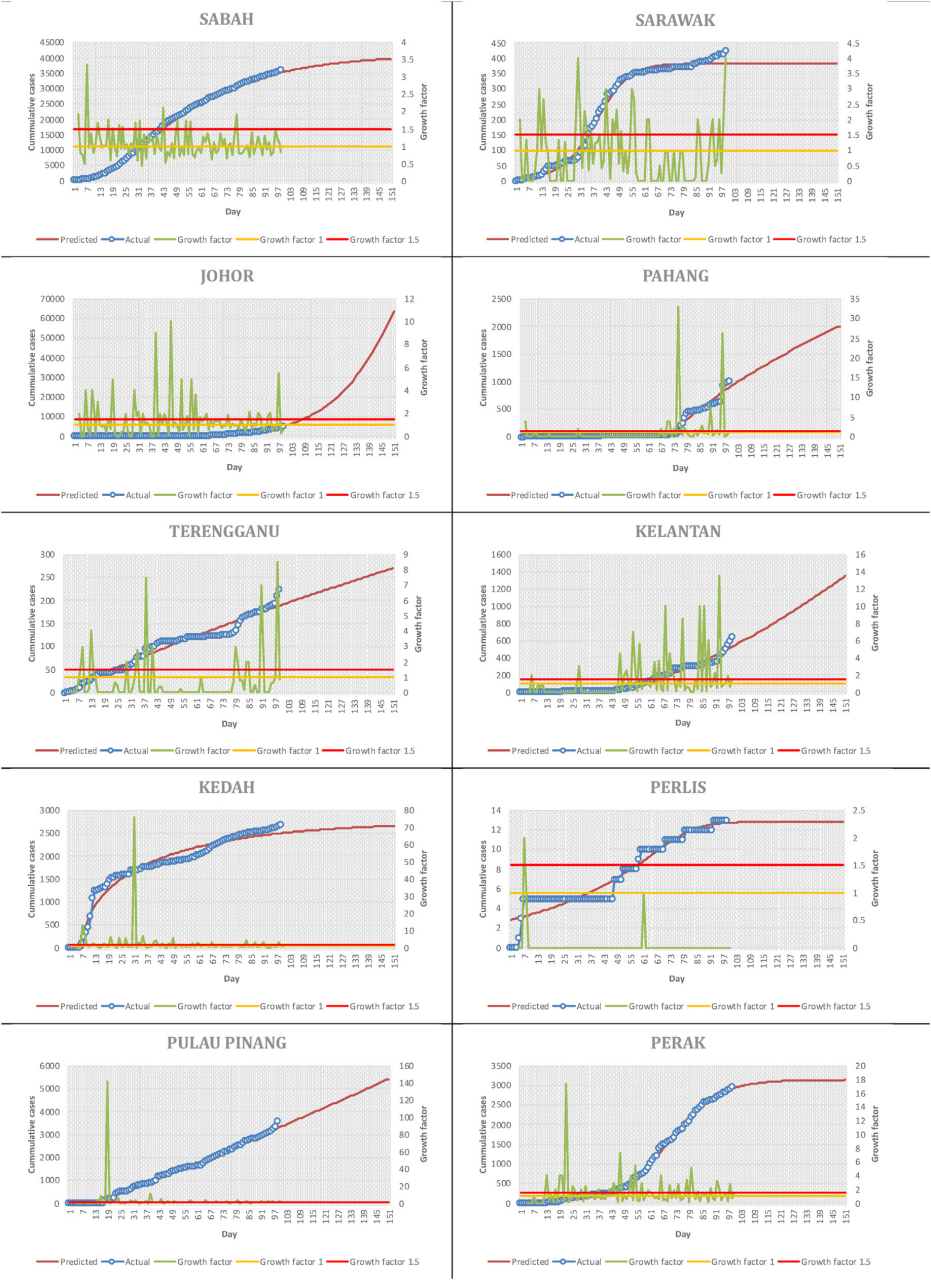

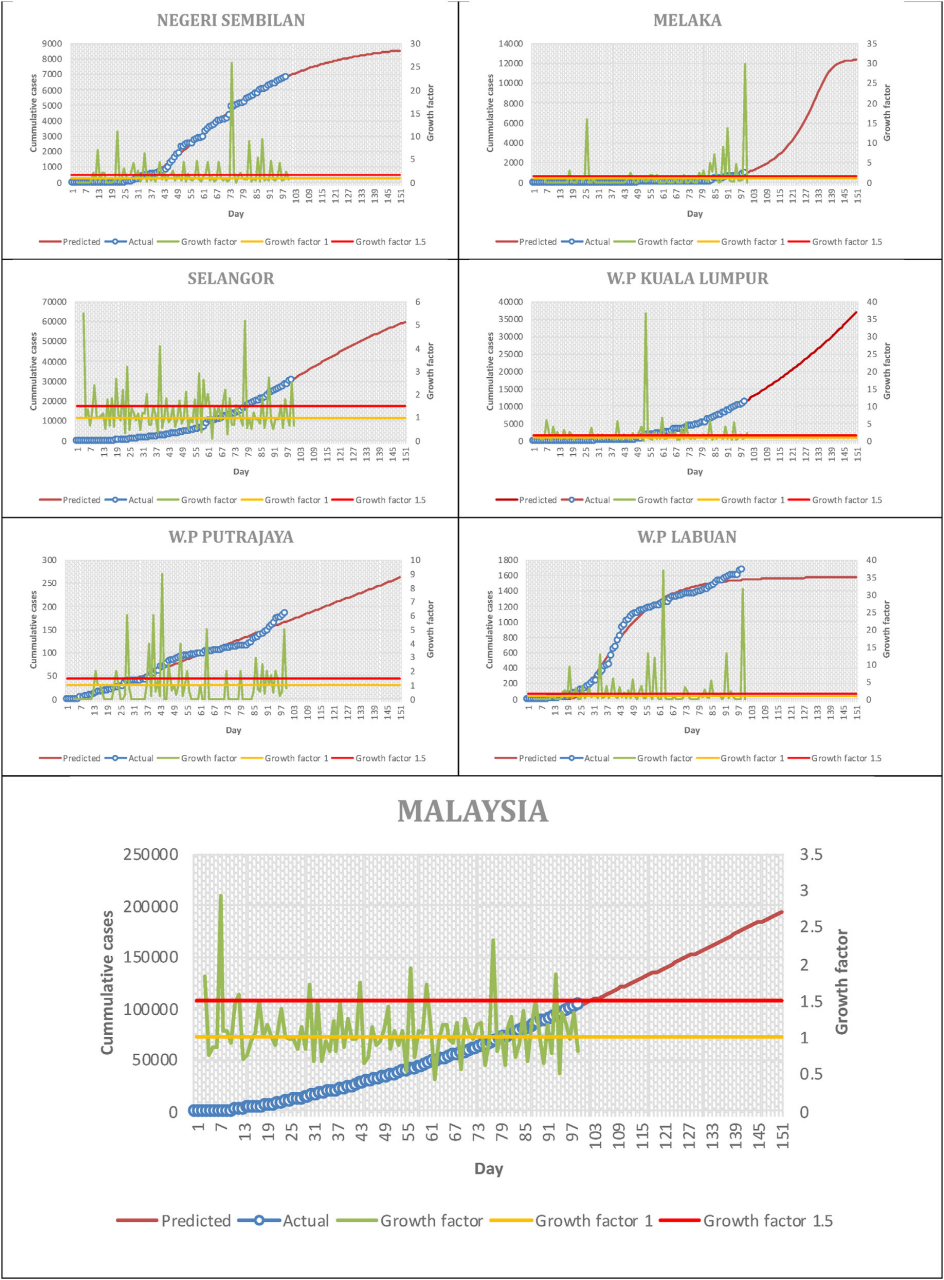
Actual cumulative cases, predicted cumulative cases, and growth factor plot by state.

Fig. 3 demonstrates the number of cases using the generalized logistic growth model, both nationally and by state. Nationally, the growth factor has been shown to range between 0.25 to a peak of 3.1 throughout the current MCO. The actual number of cases is fairly similar in trend to that predicted by the generalized logistic growth model. For the states, the 4 states with the highest number of cases – Sabah, Johor, Selangor and WP Kuala Lumpur have actual cumulative cases that mapped exactly onto the predicted cumulative cases. Terengganu, Kelantan, Sarawak and Kedah had actual cumulative cases that peaked higher than the predicted cumulative cases, with no states having less actual cumulative cases compared to predicted cumulative cases.

Table 3 demonstrates the level of the growth factor divided by 14 days intervals, corresponding with the conventional duration of each MCO extension. The growth factor range for Sabah, the state where the election-related outbreak of case figures, ranged from 1.00 to 1.25, with a narrow standard deviation of 0.22–0.72. Selangor, the other state where case numbers have remained highest throughout the MCOs, has a mean growth factor ranging from 1.22 to 1.52, with a standard deviation ranging from 0.64 to 1.25. Otherwise, the highest growth rates reported were in WP Labuan for the time periods of 22 Nov - 5 Dec 2020 and 20 Dec - 1 Jan 2021, with growth rates of 4.77 and 4.65 respectively.

**Table 3:** Growth factor every 14 days interval by state.

State		Growth factor every 14 days interval						
		27 Sep - 10 Oct 2020	11 Oct - 24 Oct 2020	25 Oct - 7 Nov 2020	8 Nov - 21 Nov 2020	22 Nov - 5 Dec 2020	6 Dec - 19 Dec 2020	20 Dec - 1 Jan 2021
SABAH	Mean	1.25	1.15	1.07	1.03	1.03	1.00	1.04
	Median	1.01	1.01	1.04	.81	1.06	.88	1.02
	Standard Deviation	.72	.39	.48	.41	.22	.36	.22
SARAWAK	Mean	1.23	.95	1.32	1.18	.93	.64	1.36
	Median	1.00	.00	1.33	1.00	1.00	.56	1.50
	Standard Deviation	.99	1.38	.78	1.01	.84	.73	1.15
JOHOR	Mean	1.49	1.38	1.76	2.22	1.17	1.10	1.41
	Median	1.00	.58	1.25	.84	1.26	.99	.97
	Standard Deviation	1.40	1.61	2.29	2.80	.48	.48	1.38
PAHANG	Mean	.75	.42	.00	.	1.41	3.74	3.24
	Median	.25	.00	.00	.	.13	1.50	.92
	Standard Deviation	1.36	.80	.00	.	1.83	8.86	7.15
TERENGGANU	Mean	1.06	.53	1.55	.12	.25	1.13	2.12
	Median	.67	.25	.40	.00	.00	.76	.67
	Standard Deviation	1.34	.78	2.38	.16	.50	1.04	3.23
KELANTAN	Mean	.64	1.00	.00	2.17	2.19	2.62	2.34
	Median	.50	.50	.00	1.00	1.24	.67	1.00
	Standard Deviation	.75	1.41	.00	2.25	2.65	3.97	3.67
KEDAH	Mean	2.70	7.03	1.90	1.47	1.32	1.24	1.11
	Median	1.21	.51	1.33	1.03	1.14	.84	.75
	Standard Deviation	3.79	19.97	1.94	1.48	.81	1.09	.96
PERLIS	Mean	1.00	.	.	.00	.33	.00	.00
	Median	1.00	.	.	.00	.00	.00	.00
	Standard Deviation	1.00	.	.	.00	.58	.	.
PULAU PINANG	Mean	1.46	11.35	2.26	1.18	1.24	1.19	1.30
	Median	.38	.79	1.19	.89	1.02	1.13	1.21
	Standard Deviation	2.56	37.35	3.08	.78	.83	.75	.64
PERAK	Mean	1.11	2.64	1.27	1.89	1.31	1.58	1.34
	Median	1.00	1.29	1.00	.90	.91	1.22	1.15
	Standard Deviation	1.27	4.44	.74	2.24	1.01	1.40	.99
NEGERI SEMBILAN	Mean	1.29	2.32	1.61	1.56	1.29	3.55	2.01
	Median	.50	1.42	1.11	1.05	.81	.93	.65
	Standard Deviation	2.08	2.99	1.73	1.45	1.38	6.85	2.64
MELAKA	Mean	.43	2.20	.20	1.08	.50	1.87	4.77
	Median	.00	1.00	.00	1.00	.00	1.00	.64
	Standard Deviation	.53	4.66	.38	.88	.76	2.15	8.62
SELANGOR	Mean	1.52	1.27	1.30	1.25	1.23	1.29	1.22
	Median	1.12	1.14	1.04	.93	1.37	.97	.94
	Standard Deviation	1.25	.85	.94	.82	.64	1.18	.73
W.P KUALA LUMPUR	Mean	1.69	1.32	1.35	3.82	1.88	1.29	1.53
	Median	.93	1.00	1.03	.98	1.14	.96	1.01
	Standard Deviation	1.68	.91	1.30	9.48	1.97	1.30	1.49
W.P PUTRAJAYA	Mean	.63	1.10	2.03	1.10	1.33	.77	1.43
	Median	.50	.50	.50	.80	.50	.25	1.00
	Standard Deviation	.69	1.84	3.01	1.19	1.97	1.13	1.31
W.P LABUAN	Mean	.35	2.09	2.38	3.00	4.77	1.28	4.65
	Median	.00	1.11	1.00	.96	.35	.84	1.00
	Standard Deviation	.67	2.57	3.39	4.36	12.13	1.52	9.65
MALAYSIA	Mean	1.21	1.11	1.05	1.04	1.05	1.07	1.11
	Median	1.00	1.00	.97	1.02	1.06	1.01	1.16
	Standard Deviation	.59	.26	.34	.34	.30	.44	.37

Table 4 shows the distribution and density of population across states in Malaysia. The most populous states are Selangor (6.5 millions), Sabah (3.9 millions) and Johor (3.7 millions), whereas the least populous are Perlis (255 thousands), WP Putrajaya (109 thousands) and WP Labuan (100 thousands). In perspective, states with higher population density include WP Kuala Lumpur, WP Putrajaya, Pulau Pinang and Selangor. On the other hand, the states with lower population density include Sarawak, Sabah, Pahang and Terengganu.

**Table 4:** Distribution of population across states in Malaysia.

State	Population (x 1000)	Area (Km^2^)	Density (Population/Area)
Selangor	6569.5	9773	672.2
Sabah	3907.5	73,631	53.1
Johor	3776.6	19,210	196.6
Sarawak	2828.7	124,450	22.7
Perak	2518.6	21,035	119.7
Kedah	2193.9	9500	230.9
Kelantan	1904.9	15,099	126.2
Pulau Pinang	1783.6	1048	1701.9
WP Kuala Lumpur	1773.9	243	7300
Pahang	1682.2	36,137	46.6
Terengganu	1259	13,035	96.6
Negeri Sembilan	1135.9	6686	169.9
Melaka	936.9	1664	563
Perlis	255	821	310.6
WP Putrajaya	108.9	49	2222.4
WP Labuan	99.6	91	1094.5

## 4 Discussion

Prediction of Covid-19 epidemiological patterns is crucial as many nations have experienced great economic privation and breakdown of social support structures in the light of the prolonged and repeated Covid-19 waves of infection (Català et al., 2020; Holmdahl & Buckee, 2020; Wang et al., 2020). Hence, it is crucial that GLM is used to produce instantaneous predictions of future trends, so that policymakers can plan accordingly (Alharbi et al., 2020; Bonnechère et al., 2021; Harvey & Kattuman, 2021). Since the GLM is trained on the existing data and is designed to fit the development of epidemic curves, rather than EID estimation only, it could provide a good fit to the limited available COVID-19 epidemiological data to characterize the transmission dynamics process and the trajectory of COVID-19 pandemic along with the impact of interventions (Wang et al., 2012). This prediction aims mainly to assess when the control measures that were implemented after the state election can result in peaking of case numbers, in order to allow swifter loosening of movement controls and permit economic activity to resume. Also, the predictions also feature abrupt variations of growth factors as well as different patterns in growth factors in different regions, as per Table 2. This is owing to the emergence of spontaneous clusters due to unchecked transmissions secondary to covert violations of the Covid-19 standard operating procedures. Hence, certain states with historically low infection numbers, e.g. WP Labuan, have sudden surges in growth factors due to the emergence of clusters. However, it is demonstrable that such growth factor surges stabilise within a few weeks, also due to the judicious use of Enhanced Movement Control Orders in Malaysia in neighbourhoods with high infectivity rates, which reduce transmissibility to other adjacent neighbourhoods and allow mass testings to be performed, weeding out pockets of asymptomatic infection.

These figures suggest that despite caseloads being high in the state of Sabah where the election took place, the growth factors stabilized rapidly. This was due to Sabah implementing much stricter movement control measures compared to the rest of Malaysia, with strict limitations of inter-district movement, shutdown of all economic sectors apart from essential services for two weeks, and restriction of opening hours of all open essential economic sectors during working hours only (Azil, 2020; Nik Anis, 2020). Inter-district travel restrictions were only loosened for a brief interregnum of one month in December 2020 in Sabah state, and were unfortunately reimposed in mid-January 2021 after a rise in case numbers in the rest of the country. In contrast, other states with higher caseloads, for instance Selangor and WP Kuala Lumpur, did not implement a strict movement control order, as most economic sectors remained open throughout October and November (Lim, 2020). The only superficial similarity in MCO in these states was the inter-district movement restriction.

Also, geographically, there was freer mixing of individuals in the 11 states and 2 federal territories of West Malaysia (all except Sabah, Sarawak and WP Labuan), as there was no requirement to have a negative Covid-19 swab before travelling to other states. This contrasted to strict entry and exit requirements imposed in Sabah, Sarawak and WP Labuan, where negative tests were required upon entry and exit (Geraldine, 2020; Ling, 2020). In Sarawak, this was compounded by a compulsory 14-day quarantine for all returning travellers, with entry and exit swabs (Chiam, 2021). This was reflected in the significantly lower case numbers and relatively low growth factors for Sarawak compared to the rest of the West Malaysian states. Also, as Sabah and Sarawak states are geographically far more isolated and larger, whereas most West Malaysian states had relatively denser population ratios and higher proportions of high rise, high density living, even though cases were no doubt higher in volume initially in Sabah state, it was more difficult for the virus to infect and disperse (Yaakub et al., 2020).

Most of the states with higher number of positive cases are predominantly states with high level of population density, such as Selangor, WP Kuala Lumpur and Johor. This finding corroborates the result of similar studies in Algeria, India, Nigeria and United States, which suggesting that the spread of COVID-19 increases in accordance with higher level of population density (Amoo et al., 2020; Babbitt et al., 2020; Bhadra et al., 2020; Kadi & Khelfaoui, 2020; Rocklöv & Sjödin, 2020; Sy et al., 2020; Yaakub et al., 2020). Peculiarly, Sabah, being the initial epicentre of the current third wave of the pandemic, also has high number of cases, despite having low level of population density. However, majority of the Sabahans reside along the coastline of Sabah instead of the interior mountainous part, and primarily concentrated over three major cities, namely Kota Kinabalu, Sandakan and Tawau (Pedersen et al., 2011). As such, these cities have far bigger and higher population density compared to other towns in Sabah, and thus, majority of the COVID-19 cases in Sabah were unsurprisingly from these three cities (Sabah State Government, 2021).

Therefore, the GLM has been key in generating short-term and long-term forecasts of the trajectory of the pandemic in various states in Malaysia with differing epidemiological risks that cover a variety of COVID-19 incidence rates. The model highlights that stricter movement control orders, such as the one that was implemented in Sabah at the early stage of the pandemic, is more effective in quickly curbing growth factors, and bringing the cases to a natural plateau earlier in the life cycle of Covid-19 transmissibility (Aziz et al., 2020; Ganasegeran et al., 2020; Tang, 2020). At the same time, there have been multiple adaptation of health care systems to the current pandemic, which require time to implement and roll out, and the strict control measures implemented in Sabah, and adoption of low-risk quarantine centers, has allowed significant slack to be cut on tertiary hospitals so that they can rightfully focus on unwell Covid-19 patients. The model also demonstrates that if MCOs are relaxed overly prematurely, what happens is that reproductive numbers and growth factors can climb prematurely (Ferguson et al., 2020; Gupta et al., 2021; Leung & Wu, 2020).

## 5 Conclusion

In conclusion, it is critical that we continue using GLM to provide governments and policymakers with accurate and helpful forecasts on magnitude of epidemic and peak time. This can assist in making long-term strategic decisions regarding procurement of Personal Protective Equipment, reagents for Covid-19 testing, and relevant medical equipment in the near and medium future, and also help governments and health ministry's plan for opening of more lower-risk facilities and decanting of non-ill patients from district hospitals where indicated in order to accommodate projected surges in admissions.

## Declaration of competing interest

The authors declare that they have no known competing financial interests or personal relationships that could have appeared to influence the work reported in this paper.
